# Supplementary material for: Quality improvement exercises in Inflammatory Bowel Disease (IBD) services: A scoping review
Source: PLoS One. 2024 Mar 7;19(3):e0298374. doi: 10.1371/journal.pone.0298374 (PMC10919633; doi:10.1371/journal.pone.0298374)
Supplement: S1 Protocol — (DOCX) [file pone.0298374.s002.docx]

**S2 Protocol. Scoping review Protocol (fixed in March 2022).**

**Quality Improvement Exercises in IBD Services: A Scoping Review**

**Background:**

Inflammatory bowel disease (IBD) is a chronic, life-long disorder in which the gastrointestinal system becomes inflamed. It is caused by the combination of the host's genetic predisposition and immune responses, as well as several environmental circumstances. Inflammatory bowel disease (IBD) includes conditions such as ulcerative colitis (UC) and Crohn's disease (CD). UC and CD were first described in 1859 (Mudler, *et al.* 2014) and 1932 (Aufses, 2001), respectively. Diarrhoea, abdominal pain, rectal bleeding, and weight loss are all signs of CD and UC. Inflammation is the prominent feature. Both disorders can strike adolescents and adults, and men and women are equally affected (Baumgart and Sandborn 2007). Managing Crohn's disease or ulcerative colitis requires more than just going to the doctor on a regular basis. It entails navigating daily activities, adhering to a treatment plan, understanding how disease affects the body and how it impacts the mind, among other things. Many IBD patients treat their condition with medication, surgery, dietary changes, and/or seeking mental support (Bannaga and Selinger 2015). IBD can be particularly difficult for people who suffer from it, it is critical to ensure the highest possible quality of treatment for patients through enhancing IBD services through quality improvement programmes, negligence to this increases the burden of healthcare as well. Studies show that IBD affects about 620 000 people in the United Kingdom. The increased prevalence of IBD, coupled with the fact that it is incurable, has major financial consequences (Gosh and Premchand 2015).

In recent years, medical treatment for IBD has vastly improved; yet, present efforts are primarily preventative rather than curative. As a result, people with IBD must deal with a lifetime condition marked by remissions and relapses. This makes IBD patients a great candidate for increased self-management in terms of care (Atreja *et al*. 2015). Our current management methods are failing us, the typical care is a little nebulous, there are many recommendations and systematic reviews (Strohl *et al.* 2018) and (Carter, Lobo and Travis 2004) to tell us what the optimum care is, but there is plenty of evidence that does not get put into practice (Danese *et al.* 2019). The psychological aspects of chronic sickness are not thoroughly thought through. A person's emotional responses to their symptoms and their sickness as a whole are referred to as negative emotional reactions. IBD can trigger unpleasant emotions such as humiliation from a bowel accident in public or anxiety of surgery (Hall et al. 2005,  Lynch and Spence 2008).

Studies from a variety of medical specialties have revealed discrepancies between the care suggested in evidence-based guidelines and the care actually provided. A high level of emergency treatment and a lack of proactive, preventative care indicate that a new strategy to service delivery is urgently needed (Crohn’s and Colitis Care in the UK, 2021). To remedy these gaps in treatment, quality improvement programmes involving systematic audit and feedback interventions, such as quality measurements, will become increasingly crucial (Shah and Hou 2014). According to Fan *et al.*  (2010), quality improvement is defined as "attempts to change clinician behaviour and, via those changes, lead to improved patient outcomes”. Everything in IBD care could be better if this call for improved services was answered. However, it is unclear what kind of information is available in literature about the quality improvement services in IBD. There hasn't been a systematic study of quality improvement strategies in IBD care yet. The goal of this study is to perform a scoping review of existing quality improvement initiatives in IBD services and to compile a list of activities that IBD practitioners can employ to improve their own practices.

**Research Question:**

What does the published literature tell us about how to improve the quality of IBD service?

**Aims:**

- To map the published quality improvement studies from IBD settings.

- To map gaps in the evidence on how IBD services can be improved.

**Objectives:**

1. A systematic search of the literature to identify quality improvement studies in the IBD setting.
2. A set of data tables that extract information on quality improvement targets, methods and outcomes.
3. A narrative synthesis and easy to digest table to guide future work.
4. A gaps analysis showing where future work is needed.

**Study Design:**

Scoping review using methods described by Arksey and O’Malley (2007). The scoping review process is especially effective for examining an emerging and diverse knowledge base, thus it is a good fit for our research question.

**Methods**

**Eligibility criteria:**

Criteria for inclusion and exclusion

The following criteria will be required for **inclusion**:

- Articles and conference abstracts that have been published in peer-reviewed publications between 2009 and 2022.
- Papers written in English
- Studies that describe at least one initiative aimed at assisting, facilitating, or improving the quality of care.
- Studies with only human patients with IBD. (Crohn's and/or Colitis)
- Studies where feasibility, acceptability, and effectiveness were evaluated prospectively or longitudinally.

The following criteria will be required for **exclusion**:

- Study based exclusively on retrospective or cross-sectional data
- Study not relevant to IBD or mixed populations (e.g. colorectal surgery)
- Study conducted in languages other than English
- Review papers
- Papers that just discuss the process of forming an initiative without gathering data about the project itself.
- Health economic studies.

**Information sources:**

MEDLINE, EMBASE, CINAHL and Web of Science are the online databases that will be searched for this scoping review. The final search results will be uploaded on Rayyan and evaluated for eligibility (see above the eligibility criteria).

**Search:**

Free text and thesaurus phrases relevant to IBD (e.g. Crohn’s Disease, ulcerative colitis, etc.) and quality improvement will be used in the search (e.g. quality management, PDSA, etc.). An example of a search technique for MEDLINE online databases can be found in

**Appendix 1.**

**Selection of sources of evidence:**

One reviewer will evaluate the titles, abstracts and then full text of publications identified by searches for potentially relevant publications. Uncertainties as to eligibility were agreed in discussion with my supervisor Dr. Dan Hind.

For each of the research selection, data charting, and critical evaluation processes, an excel sheet, as well as an explanation and elaboration document, will be generated. The documents can be internally reviewed by the supervisor if desired.

**Data Charting Process:**

This stage entails extracting data from the studies that have been included. I will work with my supervisor to create a data-charting form that will help us figure out which variables to extract to answer the research question. Charting will be an iterative procedure in which the data-charting form will be updated on a regular basis (Levac, Colquhoun and O’Brien 2010). One reviewer will independently chart the data, discuss the results and continuously update the data-charting form. This will include both general and detailed information on the study, such as the study population, kind of intervention, outcome measures used, and study design. For example, we will keep track of the following information.

**Data Items:**

1. Article characteristics:

-Publication year

-Country of origin

-Study design, based on application of the algorithm developed by Peinemann and    Kleijnen (2015)

2. Problems: for example, suboptimal vaccination rates, clinician adherence with documentation guidelines, etc.

3. Target population: children, adults or unclear

4. Number of patients and centres

5. Intervention: use of new electronic programmes, clinician education, etc

6. Clinical Assessments:  length of stay, patient satisfaction, etc

7. Findings

**Critical appraisal of individual sources of evidence:**

To characterise the methodological quality of included studies I will use the Quality Improvement Minimum Quality Criteria Set (QI-MQCS), a tool for critical appraisal of quality improvement intervention publications. The QI-MQCS was developed in collaboration with quality improvement experts to be applicable to improvement studies. It scores 16 quality criteria as met or unmet and has documented validity and reliability (Hampel *et al.* 2015).

**Synthesis of results:**

The data extracted from studies will be used to categorise them in this scoping review (see data items for extraction characteristics). The problem, intervention, and assessment approach for each study will be summarised (the categories for problem, intervention and assessment will be described in the results). This study will provide a narrative summary of quality improvement initiatives rather than a statistical summary.

**Ethical Concerns:**

Ethics is not required because this is a synthesis of published material. No human subjects are involved.

**References**

1. Aufses, A.H. Jr. (2001) ‘The history of Crohn's disease’, Surgical Clinics North America. Feb;81(1):1-11, vii. PMID: 11218157. http://doi.org/10.1016/s0039-6109(05)70270-x.

2. Arksey, H. and O'Malley, L. (2005) ‘Scoping studies: towards a methodological framework’,International Journal of Social Research Methodology, 8:1, 19-32, DOI: 10.1080/1364557032000119616

3. Bannaga, A.S. and Selinger, C.P. (2015) ‘Inflammatory bowel disease and anxiety: links, risks, and challenges faced’, Clin Exp Gastroenterol. PMID: 25848313; PMCID: PMC4376063. http://doi.org/10.2147/CEG.S57982

4. Baumgart, D.C. and Sandborn, W.J. (2007) ‘Inflammatory bowel disease: clinical aspects and established and evolving treatments’, The Lancet ;369(9573):1641–57. http://doi.org/10.1016/S0140-6736(07)60751-X

5. Carter, M.J., Lobo, A.J. and Travis, S.P.L. (2004) ‘Guidelines for the management of inflammatory bowel disease in adults’, BMJ Journals Gut ;53:v1-v16. http://dx.doi.org/10.1136/gut.2004.043372

6. Crohn’s and Colitis UK (2021) The hidden cost and vision for chance. Available at: https://s3-eu-west-1.amazonaws.com/files.crohnsandcolitis.org.uk/Health_Services/CROJ8096-IBD-National-Report-WEB-210427_(2).pdf (Accessed: 20 February 2022).

7. Danese, S. (2019) ‘Unmet Medical Needs in Ulcerative Colitis: An Expert Group Consensus’, Digestive Disease. 2019;37(4):266-283. PMID: 30726845 http://doi.org/10.1159/000496739.

8. Fan, E. et al. (2010) ‘How to Use an Article About Quality Improvement’, JAMA. 2010;304(20):2279–2287. https://doi.org/10.1001/jama.2010.1692

9. Ghosh, N. and Premchand, P.A. (2015) ‘UK cost of care model for inflammatory bowel disease’, BMJ Frontline Gastroenterol. Jul;6(3):169-174. https://doi.org/10.1136/flgastro-2014-100514.

10. Hall, N. J. et al. (2005) ‘The fight for ‘health-related normality’: A qualitative study of the experiences of individuals living with established inflammatory bowel disease (IBD)’, Journal of Health Psychology, 10(3), 443–455. http://doi.org/10.1177/1359105305051433

11. Hempel, S. et al. (2015) ‘Development of the Quality Improvement Minimum Quality Criteria Set (QI-MQCS): a tool for critical appraisal of quality improvement intervention publications’, BMJ Quality & Safety. 2015;24:796–804. https://doi.org/10.1136/bmjqs-2014-003151

12. Israel, M. and Hay, I. (2006) Research ethics for social scientists between ethical conduct and regulatory compliance. London: SAGE. (Accessed: 10 February 2022)

13. Levac, D., Colquhoun, H. and O'Brien, K.K. (2010) ‘Scoping studies: advancing the methodology’, Implementation Sci 5, 69. https://doi.org/10.1186/1748-5908-5-69

14. Lynch, T. and Spence, D. (2008) ‘A qualitative study of youth living with Crohn disease’, Gastroenterology Nursing, 31(3), 224–230. quiz 231-222. http://doi.org/10.1097/01.SGA.0000324114.01651.65

15. Ma, L. L. et al. (2020) ‘Methodological quality (risk of bias) assessment tools for primary and secondary medical studies: what are they and which is better?’, Military Medical Research, 7(1), 7. https://doi.org/10.1186/s40779-020-00238-8

16. Mulder, D.J., Noble, A.J., Justinich, C.J. and Duffin, J.M. (2014) ‘A tale of two diseases: the history of inflammatory bowel disease’, J Crohns Colitis. May;8(5):341-8. PMID: 24094598. http://doi.org/10.1016/j.crohns.2013.09.009.

17. Peinemann, F. and Kleijnen, J. (2015) ‘Development of an algorithm to provide awareness in choosing study designs for inclusion in systematic reviews of healthcare interventions: a method study’, BMJ Open. 2015;5:e007540. http://doi.org/10.1136/bmjopen-2014-007540

18. Strohl, M. et al. (2018) ’Quality of care in inflammatory bowel diseases: What is the best way to better outcomes?’, World J Gastroenterol. PMID: 29904243; PMCID: PMC6000296. http://doi.org/10.3748/wjg.v24.i22.2363.
