# Supplementary material for: Quality improvement exercises in Inflammatory Bowel Disease (IBD) services: A scoping review
Source: PLoS One. 2024 Mar 7;19(3):e0298374. doi: 10.1371/journal.pone.0298374 (PMC10919633; doi:10.1371/journal.pone.0298374)
Supplement: S1 Appendix — (DOCX) [file pone.0298374.s003.docx]

**S3 Appendix. Search strategy for scoping review on Quality Improvement initiatives in Inflammatory Bowel Diseases.**

**MEDLINE VIA OVID**

1. Inflammatory Bowel Diseases/

2. "inflammatory bowel diseas*".mp.

3. Crohn Disease/

4. "Crohn*".mp.

5. Colitis, Ulcerative/

6. "colitis".mp.

7. exp Quality Improvement/

8. exp Total Quality Management/

9. (pdsa or pdca or lean or six sigma or QI or total quality management or tqm or continuous quality management or CQM or continuous quality improvement or CQI or improvement program* or quality improv*).mp.

10. or/1-6

11. or/7-9

12. 10 and 11
