# Supplementary material for: Quality improvement exercises in Inflammatory Bowel Disease (IBD) services: A scoping review
Source: PLoS One. 2024 Mar 7;19(3):e0298374. doi: 10.1371/journal.pone.0298374 (PMC10919633; doi:10.1371/journal.pone.0298374)
Supplement: S1 Dataset — Abbreviations: US = United States; UK = United Kingdom; Paed = Paediatric; N/S = Not Stated; NA = Not Applicable; IBD = Inflammatory Bowel Disease; QI-MQCS = Quality Improvement Minimum Quality Criteria Set (critical appraisal tool); EMR = Electronic Medical Record. *Study is only available as conference abstract, not full text report. (DOCX) [file pone.0298374.s004.docx]

**S4 Dataset. Published Inflammatory Bowel Disease quality improvement studies identified in scoping review (n = 100).**

| **First Author and Year** | **Country** | **Problem Category** | **Design** | **Population** | **Patients (n)** | **Centres (n)** | **Intervention category** | **Intervention components** | **Outcome category (Process and clinical)** | **Outcomes** | **Mapped to IBD Standards** | **QI- MQCS score** |
| --- | --- | --- | --- | --- | --- | --- | --- | --- | --- | --- | --- | --- |
| Battat 2022[1] | US | Health Maintenance: Medication | Pre-post | Adult | 400 | N/S | Provider education; Feedback | Multifaceted intervention for physicians including in-person and online education and team-based audit-feedback. | Treatment rate; Adverse events | Median time to medication administration decreased, and emergency room visits before medication decreased. | Ongoing care | 7 |
| Benjamin 2023 [2] | US | Multiple quality measures | Pre-post | Adult | 73 | 1 | Patient education; New documentation; Provider education; Reminder system; Informatics | Patient questionnaire for education and to identify deficiencies; paper order sheet for health maintenance orders entered into EMR; case management log to track patients | Protocol/documentation adherence; Patient knowledge; Patient satisfaction | Ordered health maintenance items increased from; completion rate unchanged; mean patient engagement score increased. | The IBD service | 12 |
| Bensinger 2019[3] | US | Multiple quality measures | Pre-post | Adult | 150 | 2 | New documentation; Patient education; Informatics | Pre-populated note template including the Physician Quality Reporting System measures; Order set; patient education handout | Protocol/documentation adherence | Increases in documentation rates: of influenza immunisation; pneumococcal immunisations; tobacco cessation; sustained at 1 year. | IBD Service; Newly Diagnosed | 11 |
| Breton 2021[4] | US | Preventive Health: Screening - anaemia | Time series | Paed | 1628 | 1 | Informatics; New protocol; Feedback | Evidence-based care pathway created; EMR dashboard tracking anaemia metrics; automated provider monthly reports | Screening rate; Treatment rate; Remission rate | Iron deficiency screening increased; treatment rates increased; anaemia prevalence decreased. | Newly Diagnosed; Ongoing Care | 14 |
| Choe 2021[5] | US | Health Maintenance: Follow-up | Pre-post | Adult | 16 | 1 | New protocol; Provider Education; Team Change | Appointment scheduling protocol: urgent scheduling slots; dedicated IBD clinic scheduler; education to inpatient GI team | Follow-up Rate; Patient satisfaction | Mean wait time decreased from 40.4 days to 21.9 days, but change not statistically significant. Poor response to patient satisfaction survey. | Ongoing care | 12 |
| Dykes 2017[6] | US | Health Maintenance: Pre-Visit Planning compliance | Pre-post | Paed | N/S | 1 | New protocol; Informatics | Guidelines into checklist; Automated pre-visit planning checklist using EMR data | Protocol/ documentation adherence | Patients receiving complete bundle increased from 0% to approx. 100%. | Ongoing care | 13 |
| El-Matary 2016[7] | US | Multiple quality measures | Pre-post | Paed | 76 | 1 | New protocol; Patient education | Set targets to minimise steroid use and admissions; standardised assessments and investigations; scheduled visits; standardised education strategy and created handouts; Transition clinics Created IBD registry | Steroid use; Relapse; Admissions | Mean clinical relapses decreased, steroid courses decreased, emergency room visits decreased and hospital admissions decreased. | IBD Service | 8 |
| Ewelukwa 2018[8] | US | Clinician Workload | Pre-post | N/S | 824 | 1 | Team change | Incorporated scribes in clinic visits; posters and discussions about scribes | Patient satisfaction; Costs | Patient satisfaction increased from; appointment length decreased; revenue increased 536% over scribe salary. | IBD Service | 13 |
| Fofaria 2019[9] | UK | Costs/resources | Pre-post | Adult | 1083 | 3 | New protocol; Provider education | Questionnaires to gauge interest in telephone clinics; Information campaign for patients and staff | Patient satisfaction; Protocol/documentation adherence | Percentage of eligible patients transferred to telephone clinics increased; patient satisfaction remained high. | IBD Service; Ongoing Care | 14 |
| Gold 2022 [10] | US | Preventive Health: Screening - nutrition | Pre-post | Adult | 252 | 1 | New protocol; New documentation; Informatics | Screening program using modified Malnutrition Universal Screening Tool; recommendations for high risk patients; smart tool and smart phrase in EMR | Screening rate; Follow-up Rate; Adverse events; Admissions | Increase in number screened and increase in micronutrient testing for high-risk patients. Small non-significant increase in dietician referrals. | Newly diagnosed | 13 |
| Greene 2015[11] | US | Multiple quality measures | Pre-post | Adult | 200 | 20 | Provider education; Feedback | Audit feedback sessions, interactive videos, and monograph for providers | Protocol/ documentation adherence | No significant differences in quality measures, but low performing gastroenterologists showed significantly greater improvement than high performers. | IBD Service | 11 |
| Greene 2015[12] | US | Multiple quality measures | Pre-post | Adult | N/S | N/S | Provider education; Feedback | Audit feedback sessions; evidence based monographs | Protocol/ documentation adherence | Improvements of 0-48% across 9 chart measures. | IBD Service | 10 |
| Guido 2020[13] | US | Health Maintenance: Drug Monitoring | Pre-post | Paed | N/S | 1 | New protocol; Reminder system; Informatics; Provider education; Patient education | Provider education; therapy plans in EMR; parent education; pre-visit planning process; best practice alerts in EMR | Testing rate; Disease activity | Post-induction therapeutic drug monitoring increased; infliximab therapeutic drug monitoring increased; 36% of levels <5 μg/mL. | Ongoing care | 14 |
| Gupta 2019[14] | US | Health Maintenance: Medication | Pre-post | Paed | 54 | 1 | New protocol; Informatics; Provider Education; Patient Education | Offered external infusion options; standard order sets; post-infusion communication protocols; patient/provider education on options | Treatment rate | Increase in patients offered external infusions and in patients receiving external infusions. | IBD Service; Ongoing care | 12 |
| Hellmann 2021[15] | US | Health Maintenance: Drug Monitoring | Pre-post | Paed | ~230 | 2 | New protocol; Provider education; Patient education; Informatics | Provider education on care algorithm; pre-visit planning forms to alert needed labs; best practice alerts in EMR | Protocol/ documentation adherence; Remission rate | Percentage of patients with level ≥5 μg/mL and checked in 12 months improved; sustained remission improved. | Ongoing care | 13 |
| Jackson 2019[16] | Australia | Multiple quality measures | Pre-post | Adult | 100 | 2 | New protocol; Informatics | Tablet based decision support tool prior to consultations | Protocol/ documentation adherence | Increased medium use of process indicators overall from 27% to 100%, including psychological well-being management, preventive care, disease activity management; decision conflict score increased. | IBD Service; Ongoing care | 10 |
| Kaimakliotis 2021[17] | US | Health Maintenance: Medication | Pre-post | Adult | 68 | 1 | New documentation; New protocol; Provider education | Educational lecture and pocket guide on IBD pain ladder provided to residents, with re-education every 3 months | Steroid use; Length of stay; Readmission; Pain | Inpatient opioid use decreased; discharge prescriptions decreased; length of stay decreased; 90-day readmissions decreased; no significant difference in pain scores. | Flare Management; Inpatient Care | 13 |
| Kelly 2019[18] | UK | Health Maintenance: Drug Monitoring | Pre-post | Paed | 62 | 1 | New protocol; Provider education | Standard Operating Procedure for infliximab infusions; New safety checklist; Education for nurses | Protocol/ documentation adherence; Screening rate | Median safety checklist completion increased; laboratory screening adherence increased. | IBD Service; Ongoing care | 14 |
| Kozlicki 2023 [19] | US | Health Maintenance: Medication | Pre-post | Adult | 25 | 1 | Informatics | Implemented dashboard to prospectively identify outdated labs; speciality pharmacists reviewed and messaged nurses if labs required. | Treatment rate; Testing rate | Frequency of treatment gaps decreased from; median gap length decreased. | Ongoing care | 12 |
| Lambl 2019[20] | US | Preventive Health: Screening - C. difficile | Pre-post | N/S | N/S | 1 | New protocol; Provider education; Informatics | Improved environmental cleaning; guidelines and order set changes to restrict clindamycin and fluoroquinolones; modified C. diff testing algorithm | Protocol/documentation adherence; Adverse events | C. difficile rate declined; high-risk antibiotic use declined. | Inpatient Care | 13 |
| McNicol 2022 [21] | US | Preventive Health: Vaccination | Pre-post | Paed | 132 | 1 | New protocol; Provider education; Informatics | Standardised vaccination protocol and workflow in clinic and infusion centre; pre-visit planning; stakeholder engagement | Vaccination rate | Proportion of eligible patients who received hepatitis B vaccine dose 1 increased; sustained for over 12 months; proportion of patients who completed 3-dose vaccine series increased. | The IBD service | 13 |
| Melmed 2021[22] | US | Health Maintenance: Urgent Care/Triaging | Time series | Adult | 18610 | 27 | New protocol; Provider education; Patient education; Team change | Multidisciplinary care teams; urgent care slots; communication with high-risk patients; patient education on seeking urgent care | Steroid use; Admissions | System wide improvement in multiple measures: need for urgent care, hospitalisations, CT scan utilisation, steroid use, opioid use (18-50% relative reductions). | Flare Management; Ongoing care | 13 |
| Morris 2021[23] | US | Health Maintenance: Medication | Pre-post | Paed | 98 | 1 | New protocol; New documentation; Provider Education; Patient Education | Educational presentation, information sheet, process map and Frequently Asked Questions sheets for providers; clinical pharmacist checking insurance plans before anti-Tumor Necrosis Factor drugs started; patients enrolled in co-payment assistance programs | Treatment rate; Disease activity; Costs | Biosimilar greatly utilisation; no significant difference in clinical outcomes; estimated cost savings of $381,000 (average sales price) and $651,000 (wholesale acquisition cost) over 20 months | Ongoing care | 12 |
| Ong 2022 [24] | Singapore | Health Maintenance: Medication | Pre-post | Adult | 60 | 1 | Provider education; New protocol | Accelerated infliximab infusion protocol; moved infliximab collection location closer; educational interventions for physicians on eligibility criteria | Treatment rate; Costs; Adverse Events | Mean infliximab infusion time reduced; total time spent in infusion centre reduced; 3 mild infusion reactions out of 152 infusions. | The IBD service | 11 |
| Parker 2013[25] | US | Preventive Health: Vaccination | Pre-post | Adult | 84 | 1 | New documentation; Patient education | Patient education form; vaccines offered and given at same visit | Vaccination rate | Influenza vaccination increased from; pneumococcal vaccination increased. | IBD Service | 13 |
| Prendaj 2019[26] | US | Health Maintenance: Follow-up | Pre-post | Paed | 84 | 1 | New protocol; Provider education; Patient education | Phone calls to schedule appointments; Infusion visits concurrent with clinic visits; Education of physicians and patients on recommended visit frequency | Follow-up Rate | Median documented visits within 200 days increased; increase sustained for 1 year. | Ongoing care | 12 |
| Sandberg 2019[27] | US | Health Maintenance: Medication | Pre-post | Paed | 140 | 1 | New protocol; Informatics | Standardised order set for infusion orders; transitioned eligible patients to rapid infusions; eliminated post-infusion observation time; pharmacy pass-through window | Follow-up Rate | Average door-to-door time decreased; estimated 128 min per patient freed per year. | Ongoing care | 12 |
| Savarino 2016[28] | US | Health Maintenance: Follow-up | Pre-post | Paed | N/S | 1 | New protocol | Pre-visit planning for patients with upcoming visits | Remission rate | Clinical remission rate increased. | IBD Service; Ongoing care | 13 |
| Selinger 2019[29] | UK | Health Maintenance: Medication | Prospective cohort study | Adult | 2385 | 19 | New protocol; Provider education; Feedback; Patient education | Feedback of audit findings at departmental meetings; direct referral pathways from primary care; ring-fenced clinic slots for flares/urgent reviews; telephone helpline; education for clinicians and patients | Steroid use | Steroid exposure decreased; steroid excess decreased. | Flare Management; Ongoing care | 7 |
| Shah-Khan 2019[30] | US | Preventive health: Screening - Bone health | Pre-post | Adult | 65 | 1 | Provider education; Reminder system | Education lecture; flyer summarising guidelines; EMR prompt to order Bone mineral density screening | Screening rate | Bone mineral density screening rate increased. | Pre-Diagnosis | 13 |
| Shaikhalil 2018[31] | US | Health Maintenance: Enteral therapy | Pre-post | Paed | 73 | 1 | Provider education; Patient education; New Protocol; New documentation | Exclusive enteral nutrition algorithm; calorie/fluid table; provider education; talking points for family discussions; weekly rounds to review patients. | Treatment rate; Disease activity; Remission rate | Exclusive enteral nutrition utilisation increased; 71% of patients completing ≥8 weeks achieved remission; significant reduction in disease activity in patients completing. | IBD Service; Newly Diagnosed; Ongoing care | 13 |
| Shores 2019[32] | US | Preventive health: Vaccination | Pre-post | Paed | 533 | 1 | Informatics; Reminder system; Provider education | Customised prompts in EMR; education of providers about prompts and vaccination importance | Vaccination rate; Protocol/documentation adherence | Documented vaccination improved; vaccine counselling for unvaccinated patients improved. | IBD Service | 11 |
| Smith 2023 [33] | US | Preventive Health: Screening - anaemia | Pre-post | Paed (<22) | 298 | 1 | Provider education; Reminder system; Informatics; New protocol | Provider education on iron deficiency/anaemia algorithm; anaemia screening added to diagnostic checklist; EMR smart tools for ordering iron studies | Screening rate | Screening rates increased; 77% with iron deficiency/anaemia treated within 30 days. | Newly diagnosed | 13 |
| Weizman 2021[34] | US | Clinician guideline knowledge | Cluster randomised trial | Adult | 91 | 7 | Patient education | Educational video for patients summarising treatment guidelines | Screening rate; Treatment rate; Patient satisfaction; Testing rate; Length of Stay | No significant differences in length of stay or colectomy; higher trust in physician at discharge and 6 months; higher satisfaction at discharge but not sustained at 6 months; more tuberculosis skin testing within 48 hours. | Inpatient Care | 11 |
| Yogev 2021[35] | Israel | Multiple quality measures | Pre-post | Paed | 1709 | 21 | Feedback | National program with monthly feedback to 21 centres | Screening rate; Testing rate; Treatment rate; Disease activity | Increase in: obtaining anti- Tumour Necrosis Factor drug levels, faecal calprotectin utilization, bone density testing; improvement in: calprotectin <300 mg/mg, composite endpoint of inflammation resolution. | IBD Service; Ongoing care | 11 |
| *Aslam 2019[36] | US | Health Maintenance: Drug Monitoring | Pre-post | Paed | 12 | 1 | Provider education; Informatics | Implemented use of polyethylene glycol as alternative enteric contrast agent for magnetic resonance enterography | Testing rate | Polyethylene glycol better tolerated in volume, GI symptoms and willingness to drink again. | Ongoing care | 5 |
| *Axelrad 2014[37] | US | Preventive Health: Screening - C. difficile | Pre-post | N/S | 53 | 1 | Provider education | Floor nurses collected stool samples and alerted providers to order C. difficile testing for IBD inpatients | Testing rate; Adverse events; Length of stay; Readmission | Testing rate increased; tested patients more likely to have infection, shorter hospital stays and fewer readmissions. | Inpatient Care | 7 |
| *Ayers 2016[38] | US | Preventive Health: Vaccination | Pre-post | Paed | 155 | 1 | Provider education | Presented Advisory Committee on Immunization Practices recommendations at grand rounds; Chart review at 2, 8 months | Vaccination rate | Vaccine counselling improved; vaccination improved. | IBD Service | 5 |
| *Bauman 2021[39] | US | Health Maintenance: Transitional care | Pre-post | Paed | 406 | 1 | Provider education; New protocol; Reminder system | Transition readiness assessment, skills practice, goal setting and resources for ages ≥12 IBD patients | Protocol/ documentation adherence | Weekly transition service delivery improved. | IBD Service | 7 |
| *Beard 2020[40] | US | Multiple quality measures | Pre-post | N/S | N/S | 1 | New documentation; New protocol | Checklist for preventive care added to visit notes; Symptom scores to guide treatment | Protocol/ documentation adherence; Vaccination rate | Reproductive counselling, hepatitis B vaccination, DEXA scans improved - despite low checklist use. | IBD Service; Newly Diagnosed | 3 |
| *Berinstein 2021[41] | US | Health Maintenance: Care co-ordination | RCT | N/S | 425 | 1 | New protocol | Proactive disease monitoring and care co-ordination . | Costs; Quality of Life; Admissions; Adverse Events | Improved Crohn's disease-specific quality of life; no difference in charges or healthcare utilization. | NA | 7 |
| *Bhesania 2019[42] | US | Preventive health: Vaccination | Pre-post | N/S | 126 | N/S | Patient education; Reminder system | Letters, calls and emails to providers and patients about influenza vaccination | Vaccination rate | Vaccination improved. | IBD Service | 8 |
| *Bledsoe 2020[43] | Unknown | Preventive Health: VTE Prophylaxis | Pre-post | N/S | 48 | 1 | Provider education | Monthly resident lectures on inpatient IBD management including VTE prophylaxis | Treatment rate | VTE prophylaxis ordered and administered improved. | Ongoing care | 5 |
| *Bond 2018[44] | UK | Preventive Health: Pre-treatment tests | Pre-post | N/S | 18 | N/S | New protocol | Preoperative optimisation pathway for IBD surgery patients to improve modifiable risks | Protocol/documentation adherence; Length of stay; Adverse events | Compliance improved from mean 11.7 days between failures to 26.1 days; length of stay and Clavien-Dindo grade ≥II morbidity unchanged. | Flare Management | 6 |
| *Budhathoki 2021[45] | US | Preventive Health: Pre-treatment tests | Pre-post | Paed | N/S | N/S | New documentation; Informatics | Template infusion notes; order sets for infusion & labs | Testing rate | Missed labs reduced. | IBD Service | 5 |
| *Casini 2017[46] | US | Health Maintenance: Endoscopic scoring | Pre-post | Paed | N/S | 1 | New documentation; Provider education | Introduced MAYO score for UC and SES-CD score for CD; handouts, visual aids and coloured paper forms | Protocol/ documentation adherence | Scoring rates improved. | NA | 6 |
| *Caudle 2019[47] | US | Health Maintenance: Time to treatment | Pre-post | N/S | 40 | 1 | Not stated | Established and measured goals for follow-up and treatment after diagnosis | Follow-up rate; Treatment rate | Mean diagnosis to treatment decreased; sustained over 7 months. | Newly Diagnosed | 6 |
| *Chadokufa 2014[48] | UK | Multiple quality measures | Pre-post | Paed | 270 | N/S | New protocol | Enrolled patients in ImproveCareNow registry; used tools like pre-visit planner and population management report | Steroid use; Remission rate; Nutritional status | Clinical remission improved; as well as improvements in steroid free remission, patients off steroids, nutritional status, growth, nutritional failure measures. | IBD Service; Newly Diagnosed; Ongoing care | 6 |
| *Cheng 2022 [49] | US | Preventive Health: Vaccination | Pre-post | Adult | 55 | 1 | New protocol; Patient education | Called and offered nurse visits for vaccination to IBD patients on immunosuppression not up to date | Vaccination rate | Vaccinations improved for shingles, COVID-19, hepatitis B, flu, pneumococcal 23 and 13 (between 9 – 45%). | IBD Service | 7 |
| *Cho 2016[50] | US | Multiple quality measures | Pre-post | N/S | 368 | 1 | Informatics | Added computerised order set based on Crohn’s & Colitis Foundation quality indicators for IBD admissions | Treatment rate; Testing rate; Length of stay | Deep Vein Thrombosis prophylaxis improved; C. difficile testing and length of stay improved; antibiotic use decreased. | Ongoing care | 7 |
| *Coenen 2017[51] | Belgium | Clinician Workload | Pre-post | N/S | 1312 | 1 | Team change | Instituted IBD nurse position to serve as first point of contact and counsel patients | Admissions | Avoided 30 ER and 133 unscheduled clinic visits. | IBD Service | 5 |
| *Crandall 2010[52] | US | Multiple quality measures | Pre-post | Paed | 204 | 9 | New documentation; New protocol; Provider education | ImproveCareNow quality improvement collaborative including training, care algorithms, registry, pre-visit planning, population reports | Testing rate; Treatment rate; Remission rate | Increase in reliability of assessment of growth, nutrition, disease distribution and severity; remission rate increased; no significant difference in receival of recommended dose of medications. | IBD Service | 6 |
| *Danielewicz 2017[53] | US | Health Maintenance: Urgent Care/Triaging | Time series | N/S | 40 | 1 | New protocol | New call category of “IBD URGENT” to flag those with four criteria for “urgent” IBD calls | Protocol/ documentation adherence | Call volume increased as patients and staff became more familiar with the new category. | Flare Management; Ongoing care | 7 |
| *Dolan 2021[54] | US | Health Maintenance: Patient/family guidance | Time series | Paed | 76 | 1 | New protocol; Reminder system; patient education | Schedule coordination, billing code and other interventions to improve new diagnosis education attendance | Protocol/documentation adherence | Improvement in number of patients receiving all three portions of new disease education. | Newly Diagnosed | 7 |
| *Duckworth 2022 [55] | US | Preventive Health: VTE Prophylaxis | Pre-post | Paed | N/S | 1 | New protocol; Informatics | Implementation of screening tool using Epic smartphrases entered into resident daily progress notes, to risk stratify IBD admissions for venous thromboembolism prophylaxis | Protocol/documentation adherence | Daily discussion and documentation of risk improved. | Inpatient Care | 6 |
| *Fritz 2016[56] | US | Health Maintenance: Enteral Therapy | Pre-post | Paed | 396 | 1 | Informatics; Provider education; Reminder system; Feedback | Order set, education, reminders and feedback to implement nutritional surveillance guidelines | Testing rate; Protocol/ documentation adherence; Nutritional status | Increases in rate of obtaining serum iron levels, vitamin D monitoring, rates of iron/vitamin D supplementation; no significant increase in multivitamin supplementation. | IBD Service | 9 |
| *Gleeson 2023 [57] | UK | Health Maintenance: Urgent Care/Triaging | Pre-post | N/S | 1980 | 1 | New protocol | Pre-clinic screening for all patients; pathways for telephone follow-up vs in person | Follow-up Rate | IBD waiting list of 420 patients was cleared; Most expressed preference for new model. | Ongoing care | 4 |
| *Goldstein 2021[58] | US | Preventive Health: Vaccination | Pre-post | Adult | 41 | 1 | Provider education; New documentation | Training and standardised template prompts for Herpes Zoster vaccination | Vaccination rate | Vaccination improved. | IBD Service | 7 |
| *Gorti 2020[59] | US | Preventive Health: Vaccination | Pre-post | N/S | 132 | N/S | New documentation; New protocol | Embedded vaccination template in notes; Updated vaccination order menu; Created return clinic orders and nurse vaccination clinic | Vaccination rate | Vaccination rates increased for influenza, Pneuomoccocal 13 and 23, Hepatitus B, Tetanus/Diptheria/Pertussis, Herpes Zoster. | IBD Service | 9 |
| *Hans 2021[60] | US | Health Maintenance: Drug Monitoring | Pre-post | N/S | 71 | 1 | Informatics; Reminder system | Used IBD dashboard to identify overdue patients; GI PharmD notified nurse manager of overdue patients; Nurse manager called patients; MD/PharmD renewed labs | Testing rate | Patients up-to-date on labs increased. | IBD Service | 10 |
| *Hasan 2022 [61] | US | Preventive Health: Screening - social determinants of health | Pre-post | Paed | 320 | 1 | New protocol | Offered 8-item social determinants of health screener in 9 languages; provided resource sheet for positive screens | Screening rate | Social determinants of health screening rate per week improved. Of those screened, 17.4% were positive for at least 1 risk factor. | Ongoing care | 5 |
| *Huggett 2014[62] | UK | Multiple quality measures | Pre-post | Paed | 270 | N/S | New protocol | Adopted ImproveCareNow standards and tool; monthly and weekly to review goals and discuss individual treatment plans | Remission rate; Nutritional status; Disease activity | Remission rates increased; steroid-free remission increased; nutritional status increased; growth status increased; nutritional failure decreased; Mild disease activity decreased; moderate to severe disease decreased. | NA | 9 |
| *Hyams 2022 [63] | UK | Preventive Health: Screening - bone health | Pre-post | N/S | 179 | 1 | Provider education; Informatics | Education session on guidelines; Created electronic screening tool | Screening rate | Screening improved for smoking and exercise but not alcohol; small non-significant improvement in DEXA scans. | Ongoing care | 4 |
| *Iqbal 2019[64] | US | Preventive Health: Vaccination | Pre-post | N/S | 63 | 1 | Provider education | Educational session for fellows and nurse practitioners | Vaccination rate | Fellows' vaccination rates increased for zoster, pneumovax, hepatitis A and B; Nurse practitioners' rates increased for zoster, influenza and pneumonia vaccines. | IBD Service | 4 |
| *Juakiem 2018[65] | US | Multiple quality measures | Pre-post | Adult | 275 | 1 | New documentation; Provider Education; Reminder system | In-service education; standardised template with built-in quality measures | Protocol/ documentation adherence | Overall compliance with IBD health maintenance score increased; significant improvement in 9 of 15 measures; but compliance score dropped slightly in year 2 and 3. | NA | 8 |
| *Keith 2018[66] | Unknown | Preventive Health: Vaccination | Pre-post | Paed | 119 | 1 | New protocol; Reminder system | Offered influenza vaccine in IBD clinic; provider reminder to recommend vaccine in pre-visit planning meetings | Vaccination rate; Protocol/ documentation adherence | Influenza vaccination increased; documentation of vaccine status increased. | IBD Service | 7 |
| *Koumoutsos 2018[67] | UK | Health Maintenance: Stratified care: Primary sclerosing cholangitis | Pre-post | Adult | 47 | 1 | Provider education; New protocol | Reiteration to physicians at clinics to actively identify patients; prospective database maintained; patients discussed in hepatology and regular IBD multidisciplinary meetings; joint clinic established | Treatment rate; Screening rate | Improved annual surveillance with magnetic resonance cholangiopancreatography, colonoscopy, chromoendoscopy, and MRI liver; 49% of patients had changed management after meetings; led to diagnoses and treatment. | IBD Service | 7 |
| *Lee 2014[68] | US | Multiple quality measures | Pre-post | N/S | N/S | 1 | Provider education | Educational session for fellows on IBD quality measures | Protocol/ documentation adherence | Improvement in documentation/recommendation compliance for hepatitis A/B, influenza, pneumococcus, HIV, skin cancer, and depression. | Inpatient Care | 5 |
| *Lendner 2022 [69] | US | Health Maintenance: Medication | Pre-post | Paed | 13 | 1 | New protocol | Weekly nurse phone calls for patients on steroids; standardised wean schedule; dot phrase for documentation | Follow-up Rate; Steroid use; Admissions | Increased number of patients who were contacted within 2 weeks to evaluate steroid wean, and number weaned off by 8 weeks; no increase in admissions for flares. | Ongoing care | 5 |
| *Levine 2019[70] | Unknown | Preventive Health: VTE Prophylaxis | Observational cohort | Paed | 234 | 1 | Informatics | Risk stratification algorithm to identify high risk of venous thromboembolism, incorporated into EMR | Treatment rate; Adverse events | Electronic screened patients more likely to receive enoxaparin; no bleeding complications | Flare Management | 5 |
| *Lin 2022 [71] | Singapore | Preventive Health: Vaccination | Pre-post | N/S | 246 | 1 | New protocol; Patient education; Team Change | Vaccination guidelines protocolised; information pamphlet for patients; nurse screening; counselling; in-clinic vaccination | Vaccination rate; Protocol/documentation adherence | Influenza and pneumococcal vaccination increased; vaccination documentation increased | The IBD service | 6 |
| *Maisa 2014[72] | US | Multiple quality measures | Pre-post | Adult | 105 | 1 | Informatics; Reminder System | Interactive flow sheet in EMR to provide reminders about screening and monitoring, with smart phrases | Testing rate; Vaccination rate; Screening rate | Documenting numerous preventive health care measures increased; improved vaccination rates; bone density testing not improved. | Ongoing care | 8 |
| *Mansoor 2017[73] | US | Health Maintenance: Enteral therapy | Pre-post | Paed | 10 | 1 | New protocol; Patient education; informatics | Enteral nutrition protocol for induction/maintenance; videos and education session for families; standardised orders and instructions | Remission rate | Of 7 who completed induction, BMI improved 16.1 to 17. | Ongoing care | 9 |
| *Mathew 2019[74] | US | Preventive health: Vaccination | Pre-post | Paed | 122 | 1 | New protocol | Mailers, calls and provider notification to patients; offered vaccine during visits | Vaccination rate | Influenza vaccination increased | IBD Service; Ongoing care | 6 |
| *McConnell 2017[75] | US | Multiple quality measures | Pre-post | N/S | 16 | 1 | Provider education; New documentation; Informatics | Standardised initial consult text; checklist for subsequent notes; order set; education for residents/hospitalists; narcotics awareness campaign | Testing rate; Length of stay; Vaccination rate | Preliminary results show improved performance for *C. difficile* testing, VTE prophylaxis ordered, colorectal surgery consultations. | Inpatient Care | 8 |
| *Morency 2022 [76] | US | Preventive Health: VTE Prophylaxis | Pre-post | Paed | 28 | 1 | Provider education; New protocol | Guidelines to identify patients at risk; dot phrase in EMR for consultation need, incorporated into general history and physical note template | Protocol/documentation adherence; Treatment rate | Dot phrase use increased. | Inpatient Care | 7 |
| *Navarro 2022 [77] | US | Multiple quality measures | Pre-post | N/S | 252 | 1 | Reminder system; Informatics; New protocol | Email, text message, and EMR reminders for data collection and input into Improve Care Now registry; one-by-one review of patient records | Protocol/documentation adherence | Data collection and input into registry improved. | The IBD service | 3 |
| *Pasquarella 2017[78] | US | Health Maintenance: Patient/family guidance | Pre-post | Paed | 50 | N/S | Patient education | Education via disease information health cards | Patient knowledge | Significant improvement in patients' disease knowledge scores but not parents' scores. | Ongoing care | 6 |
| *Patel 2021[79] | US | Health Maintenance: Stratified care: Obesity | Pre-post | Paed | 173 | 1 | Provider education; Reminder system | Screening algorithm; multidisciplinary meetings; virtual nutrition classes; EMR tools and reminders; patient handouts | Screening rate | HbA1c and Lipid panels in overweight/obese patients increased; dietician visits increased. | Ongoing care | 7 |
| *Penninti 2019[80] | US | Preventive Health: VTE Prophylaxis | Pre-post | N/S | 18 | 1 | Reminder system | Reminder sheet on prophylaxis parameters; email to providers | Treatment rate; Costs | VTE prophylaxis rates improved. | Flare Management | 8 |
| *Perlman 2019[81] | US | Multiple quality measures | Pre-post | Paed | N/S | 1 | Provider education; Reminder system, New documentation | Insertion of Paediatric Ulcerative Colitis Activity Index section in notes; division teaching session; monthly email reminders | Protocol/documentation adherence | Documentation increased; Adherence to treatment guidelines on days 3 and 5 increased. | Inpatient Care | 7 |
| *Prendaj 2017[82] | US | Preventive Health: Screening - Vitamin D | Pre-post | Paed | 94 | 1 | Provider education; Feedback; Informatics | Education; emails on performance; order set creation | Screening rate | Vitamin D screening increased. | NA | 7 |
| *Raj 2019[83] | US | Preventive Health: Mental health | Pre-post | N/S | 56 | 1 | New protocol | Creation of Standard Operating Procedure; staff training; materials created in Spanish | Screening rate | Depression screening rate increased. | NA | 7 |
| *Reich 2014[84] | US | Preventive Health: Vaccination | Pre-post | N/S | 1593 | 1 | New protocol; Patient education | One-page education form for patients on importance of vaccines; offered vaccines during visits | Vaccination rate | Influenza vaccination increased; Pneumococcal vaccination increased. | IBD Service | 9 |
| *Rudra 2021[85] | US | Preventive Health: Vaccination | Pre-post | Paed | 60 | 1 | Provider education; New protocol; Informatics | Educational sessions; EMR order set; phone screening and vaccine offering during appointment | Vaccination rate | Vaccination rate increased. | IBD Service | 8 |
| *Saffouri 2015[86] | US | Preventive health: Screening - bone health | Pre-post | Adult | 150 | 1 | Patient education | Informational patient handouts | Screening rate | No significant difference in bone health discussion or Bone Density Evaluation recommendation. | Newly Diagnosed | 7 |
| *Salem 2020[87] | US | Multiple quality measures | Pre-post | N/S | 35 | 1 | New documentation | Simple template for quality metrics added to clinic notes | Protocol/ documentation adherence | Overall adherence to ‘Bridges to Excellence’ program increased. | Ongoing care | 5 |
| *Scherl 2019[88] | US | Health Maintenance: Time to treatment | Pre-post | Adult | N/S | 22 | Feedback | Audit-feedback; action plans to reduce delays in access to biologics | Treatment rate | Days decreased between prescription request and prior authorisation, prescription written, and prescription dispensed. | Ongoing care | 2 |
| *Shakweh 2021[89] | UK | Health Maintenance: Endoscopic scoring | Pre-post | N/S | N/S | 1 | Provider education; Informatics | Proforma integrated into reporting software; training on endoscopic indices; posters | Screening rate | Use of indices increased. | Ongoing care | 3 |
| *Singh 2023 [90] | UK | Health Maintenance: Treat to Target | Pre-post | N/S | 7934 | 38 | Provider education; Feedback | Monthly performance reports; webinars; learning sessions | Protocol/documentation adherence; Remission rate | Rate of 'intention to treat to target' increased; trends towards increased remission. | The IBD service | 4 |
| *Smith 2021[91] | US | Preventive health: Screening - iron deficiency | Pre-post | Paed | 154 | 1 | Provider education; Reminder system | Algorithm for anaemia screening and treatment; provider education; EMR reminders and enhancements | Screening rate; Treatment rate | Screening increased; treatment within 2 weeks from lab result remained similar. | Newly Diagnosed | 7 |
| *Subramanian 2022 [92] | US | Preventive Health: Screening - bone heath | Pre-post | Adult | 85 | 1 | New documentation; Provider education | Providers education on importance of bone health maintenance; smart phrase in EMR | Screening rate; Adverse events | Bone density scan screening increased; vitamin D monitoring increased | Ongoing Care | 6 |
| *Sussman 2022 [93] | US | Health Maintenance: Patient/family guidance | Pre-post | Paed | 5 | 1 | Patient education | 20-minute biologic education session for patients and caregivers | Patient Knowledge; Treatment rate | Increased patient knowledge of medication shown in surveys. | The IBD service | 3 |
| *Ta 2021[94] | US | Health maintenance: Medication | Pre-post | Paed | 184 | 1 | Provider education; Reminder system | Tailored staff education on target adalimumab levels; personalised emails of specific patients; EMR best practice alert on low levels; updated EMR sheets to include timing of labs | Treatment rate; | Improved adalimumab trough drug levels. | Ongoing care | 9 |
| *Talmadge 2020[95] | Unknown | Preventive health: Mental health | Pre-post | Paed | 141 | 1 | New protocol; Provider education | Staff training on screening tools; streamlining of processes | Screening rate | Weekly screening rates improved. | NA | 8 |
| *Tse 2021[96] | US | Health maintenance: Medication | Pre-post | Adult | 74 | 1 | Informatics; Provider education | Standardised 6-week prednisone taper order set; house staff education on problem of chronic steroid use | Steroid use | Rate of decreasing steroid dose at 3 months post-discharge increased; high dose steroid use decreased. | Ongoing care | 5 |
| *Turner 2019[97] | Israel | Multiple quality measures | Pre-post | Paed | 1657 | 20 | Feedback | Monthly performance reports | Disease activity | Increase in biologic use; Increase in measures of mucosal healing. | NA | 9 |
| *Vigano 2016[98] | Italy | Multiple quality measures | Pre-post | N/S | N/S | Unclear | Provider education | Conference and educational materials on quality indicators | Protocol/documentation adherence | Improvements seen in some preventative infectious disease practices, but not all. | NA | 3 |
| *Walker 2019[99] | US | Preventive Health: Vaccination | Pre-post | N/S | 308 | 1 | Provider education; New protocol | Clinical protocol requiring vaccination inquiry and recommendation at each visit | Vaccination rate | Vaccination adherence increased for influenza, pneumococcal, and hepatitis B vaccines | IBD Service | 6 |
| *Walker 2019[100] | Unknown | Health Maintenance: Drug Monitoring | Pre-post | N/S | 308 | 1 | New protocol; Provider education | Established clinical protocols for therapeutic drug monitoring; educational sessions | Protocol/documentation adherence; Steroid use; Adverse events | Therapeutic drug monitoring increased for biologics and thiopurines; also associated with decreased steroid use and decreased IBD-specific surgery | Ongoing care | 3 |

Abbreviations: US = United States; UK = United Kingdom; Paed = Paediatric; N/S = Not Stated; NA = Not Applicable; IBD = Inflammatory Bowel Disease; QI-MQCS = Quality Improvement Minimum Quality Criteria Set (critical appraisal tool); EMR = Electronic Medical Record.

*Study is only available as conference abstract, not full text report.

**References**

1. Battat R, Galati JS, Lukin D, Chabouni F, Sockolow R, Carter J, et al. A Quality Improvement Initiative Is Associated With Reduced Time to Administer Biologics and Small Molecules and Emergency Room Visits in Inflammatory Bowel Disease. Journal of Clinical Gastroenterology. 2022;56(3):e176–82.

2. Benjamin L.E., Gianelis K.A. Using American College of Gastroenterology Guidelines to provide effective health maintenance for patients with inflammatory bowel disease. J Am Assoc Nurse Pract. 2023;35(1):86–92.

3. Bensinger A, Wilson F, Green P, Bloomfeld R, Dharod A. Sustained Improvement in Inflammatory Bowel Disease Quality Measures Using an Electronic Health Record Intervention. Appl Clin Inform. 2019;10(05):918–26.

4. Breton J, Witmer CM, Zhang Y, Downing M, Stevenson J, McDermott J, et al. Utilization of an Electronic Medical Record-integrated Dashboard Improves Identification and Treatment of Anemia and Iron Deficiency in Pediatric Inflammatory Bowel Disease. Inflamm Bowel Dis. 2021;27(9):1409–17.

5. Choe MY, VanGraafeiland B, Parian A. Improving Follow-ups With Gastroenterologists Utilizing an Appointment Scheduling Protocol in Inflammatory Bowel Disease: A Quality Improvement Project. Gastroenterology Nursing. 2021;44(5):E91–100.

6. Dykes DMH, Jean MR, Morgan P, Hill D, Williams E, Opipari-Arigan L, et al. Improving health maintenance supervision in a paediatric IBD clinic. BMJ Open Qual. 2017;6(2):e000012.

7. El-Matary W, Dufault B. Quality improvement in paediatric inflammatory bowel disease: the Manitoba experience. Acta Paediatr. 2016;105(9):e440–2.

8. Ewelukwa O, Perez R, Carter LE, Fernandez A, Glover S. Incorporation of Scribes Into the Inflammatory Bowel Disease Clinic Improves Quality of Care and Physician Productivity. Inflamm Bowel Dis. 2018;24(3):552–7.

9. Fofaria RK, Barber S, Adeleke Y, Woodcock T, Kamperidis N, Mohamed A, et al. Stratification of inflammatory bowel disease outpatients by disease activity and risk of complications to guide out-of-hospital monitoring: a patient-centred quality improvement project. BMJ Open Qual. 2019;8(3):e000546.

10. Gold S, Kohler D, Philippou A, Rabinowitz L, Manning L, Keefer L, et al. Feasibility and impact of a quality improvement initiative to screen for malnutrition in an Inflammatory Bowel Disease clinic. CLINICAL NUTRITION ESPEN. 2022 Dec;52:371–6.

11. Greene L, Sapir T, Moreo K, Carter JD, Patel B, Higgins PDR. Impact of Quality Improvement Educational Interventions on Documented Adherence to Quality Measures for Adults with Crohnʼs Disease: Inflammatory Bowel Diseases. 2015;21(9):2165–71.

12. Greene L, Moreo K. Quality improvement education to improve performance on ulcerative colitis quality measures and care processes aligned with National Quality Strategy priorities. BMJ Qual Improv Report. 2015;4(1):u208829.w3554.

13. Guido AJ, Crandall W, Homan E, Dotson J, Maltz RM, Donegan A, et al. Improving Post-induction Antitumor Necrosis Factor Therapeutic Drug Monitoring in Pediatric Inflammatory Bowel Disease. Journal of Pediatric Gastroenterology & Nutrition. 2020;70(1):48–54.

14. Gupta SR, Crandall WV, Donegan A, Johnson M, Drobnic B, Oates M, et al. A Quality Improvement Approach to External Infliximab Infusions in Pediatric Inflammatory Bowel Disease. Journal of Pediatric Gastroenterology & Nutrition. 69(5):544–50.

15. Hellmann J, Etter RK, Denson LA, Minar P, Hill D, Dykes DM, et al. Quality Improvement Methodology Optimizes Infliximab Levels in Pediatric Patients with Inflammatory Bowel Disease. Pediatric Quality & Safety. 2021;6(3):e400.

16. Jackson B, Begun J, Gray K, Churilov L, Liew D, Knowles S, et al. Clinical decision support improves quality of care in patients with ulcerative colitis. Aliment Pharmacol Ther. 2019;49(8):1040–51.

17. Kaimakliotis P, Ramadugu A, Kang J, McGorisk T, Polick A, Votta-Velis E, et al. Targeted housestaff intervention reduces opioid use without worsening patient-reported pain scores and improves outcomes among patients with IBD: the “IBD pain ladder”. Int J Colorectal Dis. 2021;36(6):1193–200.

18. Kelly MM, Turner BS, Kappelman MD, Lee EJ, Gulati AS. Implementation and Evaluation of a Standard Operating Procedure for Pediatric Infliximab Infusions. Pediatric Quality & Safety. 2019;4(1):e137.

19. Kozlicki M, Lynch B, Donoho T, Nichols P, Zuckerman AD. Development and implementation of a laboratory monitoring dashboard to reduce treatment gaps in inflammatory bowel disease. Am J Health-Syst Pharm. 2023;80(Supplement_2):S55–61.

20. Lambl BB, Altamimi S, Kaufman NE, Rein MS, Freeley M, Duram M, et al. Leveraging Quality Improvement Science to Reduce C. difficile Infections in a Community Hospital. The Joint Commission Journal on Quality and Patient Safety. 2019;45(4):285–94.

21. McNicol M, Donegan A, Hawa K, Boutzoukas A, Drobnic B, Oates M, et al. Improving Hepatitis B Vaccination Rates among At-risk Children and Adolescents with Inflammatory Bowel Disease. PEDIATRIC QUALITY & SAFETY. 2022 Jul;7(4).

22. Melmed GY, Oliver B, Hou JK, Lum D, Singh S, Crate D, et al. Quality of Care Program Reduces Unplanned Health Care Utilization in Patients With Inflammatory Bowel Disease. Am J Gastroenterol. 2021;116(12):2410–8.

23. Morris GA, McNicol M, Boyle B, Donegan A, Dotson J, Michel HK, et al. Increasing Biosimilar Utilization at a Pediatric Inflammatory Bowel Disease Center and Associated Cost Savings: Show Me the Money. Inflammatory Bowel Diseases. 2022;28(4):531–8.

24. Ong W, Lim M, Chan E, Lim T, Lim T, Chan W. A quality improvement project reduces time spent at an inflammatory bowel disease infusion center with accelerated infliximab infusion protocol. JGH OPEN. 2022 Jul;6(7):470–6.

25. Parker S, Chambers White L, Spangler C, Rosenblum J, Sweeney S, Homan E, et al. A Quality Improvement Project Significantly Increased the Vaccination Rate for Immunosuppressed Patients with IBD: Inflammatory Bowel Diseases. 2013;19(9):1809–14.

26. Prendaj E, Thomas S, Tomer G. Population Management: A Tool to Improve Timely Care in Pediatric and Young Adult Patients with Inflammatory Bowel Disease. Gastroenterology Research and Practice. 2019;2019:1–7.

27. Sandberg KC, Lucien JN, Stoll D, Yanney E, Mezoff A. Decreasing Door-to-Door Times for Infliximab Infusions in a Children’s Hospital Observation Unit. Pediatr Qual Saf. 2019;4(1):e131.

28. Savarino JR, Kaplan JL, Winter HS, Moran CJ, Israel EJ. Improving Clinical Remission Rates in Pediatric Inflammatory Bowel Disease with Previsit Planning. BMJ Quality Improvement Reports. 2016;5(1):1–5.

29. Selinger CP, Parkes GC, Bassi A, Limdi JK, Ludlow H, Patel P, et al. Assessment of steroid use as a key performance indicator in inflammatory bowel disease-analysis of data from 2385 UK patients. Aliment Pharmacol Ther. 2019;50(9):1009–18.

30. Shah-Khan SM, Cumberledge J, Shah-Khan SM, Gannon K, Kupec JT. Improving bone mineral density screening in patients with inflammatory bowel disease: a quality improvement report. BMJ Open Qual. 2019;8(3):e000624.

31. Shaikhkhalil AK, Boyle B, Smith J, Dotson JL, Donegan A, Kim SC, et al. Using Quality Improvement to Increase Utilization of Enteral Therapy in Pediatric Crohn Disease: Results and Outcomes. Journal of Pediatric Gastroenterology & Nutrition. 2018;66(6):909–14.

32. Shores D, Wilson L, Oliva-Hemker M. Utilizing Information Technology to Improve Influenza Vaccination in Pediatric Patients With Inflammatory Bowel Disease: Gastroenterology Nursing. 2019;42(4):370–4.

33. Smith J, Jacobson-Kelly A, Donegan A, Boyle B, Maltz RM, Michel HK, et al. Diagnosis and Treatment of Iron Deficiency and Anemia in Youth With Inflammatory Bowel Disease. J Pediatr Gastroenterol Nutr. 2023;76(3):313–8.

34. Weizman AV, Bressler B, Seow CH, Afif W, Afzal NM, Targownik L, et al. Providing Hospitalized Ulcerative Colitis Patients With Practice Guidelines Improves Patient-Reported Outcomes. Journal of the Canadian Association of Gastroenterology. 2021;4(3):131–6.

35. Yogev D, Shosberger A, Nehemia C, Harel S, Yerushalmy-Feler A, Ledder O, et al. Monitoring Enables Progress: A Nationwide Quality Improvement Program in Children With Crohn’s Disease. Journal of Pediatric Gastroenterology & Nutrition. 2021;73(2).

36. Aslam S, Abraham J, Bradley G. Improving utilization of proactive anti-TNF therapeutic drug monitoring: a quality improvement initiative. Journal of Pediatric Gastroenterology and Nutrition. 2019;69(Sup 2).

37. Axelrad JE, Shah BJ. Clostridium difficile Infection in Inflammatory Bowel Disease: A Nursing-Based Quality Improvement Strategy. J Healthc Qual. 38(5):283–9.

38. Ayers M, Mahajan L, Anani A, Collyer E. Immunization rates for PPSV23 (Pneumovax) in immunocompromized pediatric patients with inflammatory bowel disease: room for improvement. Journal of Pediatric Gastroenterology and Nutrition. 2016;63:Sup 2.

39. Bauman L, Hemperly A, Choi L, Yu E, Nidhi G, Kumar S, et al. Improving transition service delivery to adolescents and young adults with inflammatory bowel disease at an academic medical center. Journal of Pediatric Gastroenterology and Nutrition. 2021;73(S1):S81–3.

40. Beard JI, Kahloon A. Slowly but Surely: One Clinic’s Path to Improvement in the Health Maintenance Quality Measures of the Inflammatory Bowel Disease Population. Official journal of the American College of Gastroenterology | ACG. 2020;115:S407.

41. Berinstein J, Greenberg G, Cohen-Mekelburg S, Higgins PD. Clinical coordination and intense proactive monitoring to improve utilization of resources and reduce expenditures in high-risk IBD patients (CAPTURE IBD): A randomized, controlled quality improvement trial. Gastroenterology. 2021;160(6):S-156-S-157.

42. Bhesania N, Mahajan L, Cohen M, Kaplan B. Improving influenza vaccination rate in patients with inflammatory bowel disease on adalimumab: A quality improvement project. Journal of Pediatric Gastroenterology and Nutrition. 2019;69(Sup 2).

43. Bledsoe AC, Quinn K, Genere JR, Hansel SL, Raffals LH, Kane SV. Improving venous thromboembolism prophylaxis in patients hospitalized with inflamatory bowel disease: A quality improvement initiative. Gastroenterology. 2020;158(6):S-108.

44. Bond A, Davies K, Stansfield C, Owen K, Lal S, Soop M. Optimisation of patients prior to IBD related resection using a quality improvement methodology. Gut. 2018;67(Sup 1):A231–A231.

45. Budhathoki R, Forges-Voigt C, Sosa M, Langshaw A. Improving compliance to important laboratory work during biologic infusion: A quality improvementproject. Journal of pediatric gastroenterology and nutrition. 2021;73:S91–2.

46. Casini R, Blaufuss T, Reddy M. Endoscopic scoring to assist in the management of pediatric inflammatory disease. Journal of Pediatric Gastroenterology & Nutrition. 2017;65(2):S1–359.

47. Caudle D, Yarger E, Abernathy LY, Sandberg K. Diagnosis to treatment of inflammatory bowel disease. Journal of Pediatric Gastroenterology and Nutrition. 2019;69(Sup 2).

48. Chadokufa S, Huggett B, Kiparissi F, Terry S, Shah N, Lindley K, et al. ImproveCareNow (ICN) as a quality improvement (QI) tool in a paediatric inflammatory bowel disease (pIBD). Journal of Crohn’s and Colitis. 2014;8:S405.

49. Cheng T, Kreitman K, Stowe C, Waters B, Baidoo L. Improving the vaccination rates for immunosuppressed patients with inflammatory bowel disease. Gastroenterology. 2022;162(3):S84–5.

50. Cho J, Feder R, Rowan K, Chancay M, Fazzari M. Educational Value of an IBD Inpatient Order Set. The American Journal of Gastroenterology. 2016;111(Sup 1):S330–1.

51. Coenen S, Weyts E, Vermeire S, Ferrante M, Noman M, Ballet V, et al. Effects of introduction of an inflammatory bowel diseases nurse position on the quality of delivered care. Journal of Crohn’s and colitis. 2016;10(Sup 1):S497–8.

52. Crandall W, Kappelman M, Colletti RB, Denson L, Duffy LF, Grunow J, et al. Improved Outcomes in a Quality Improvement Collaborative for Pediatric Ulcerative Colitis. Gastroenterology. 2010;138(5):S-29.

53. Danielewicz M, Shah SA. Enhancing Access to Urgent Care in IBD in a Community Practice: 672. American Journal of Gastroenterology. 2017;112:S374.

54. Dolan C, Mudarri S, Hirsch R, Jonas M, Dybowski S, Arnold J, et al. Improving multidisciplinary education for patients with newly diagnosed inflammatory boweldisease (IBD). Journal of pediatric gastroenterology and nutrition. 2021;73:S340–1.

55. Duckworth L., Molleston J., Tica S., Davis T., Saini S., Samson C. A quality improvement project to prevent venous thromboembolic events in inflammatory bowel disease. J Pediatr Gastroenterol Nutr. 2022;75(Supplement 1):S233–4.

56. Fritz, Julia, Cabrera, Jose, Walia, Cassandra. Improving nutritional surveillance and supplementation in children with inflammatory bowel disease. Journal of Pediatric Gastroenterology & Nutrition. 2016;63(2):S1–415.

57. Gleeson S., Buckley P.M., McCarthy D.J., Sugrue K., O’Grady D.J., O’Sullivan C., et al. Development and introduction of a pre-clinic screening, triage system and virtual consultations for patients with Inflammatory Bowel Disease: A nurse led quality improvement project (QIP). J Crohn’s Colitis. 2023;17(Supplement 1):i1046–7.

58. Goldstein RS, Bhatt A. Increasing Herpes Zoseter Vaccination Rates in a High Risk IBD Population. Gastroenterology. 2021;160(6):S-86.

59. Gorti H, Shelnut D, Prasad M. Improving Vaccination Rates in Patients With Inflammatory Bowel Disease. Official journal of the American College of Gastroenterology | ACG. 2020;115:S636.

60. Hans AK, Farino V, Nwafor B, Laisamma J, Prasad M. A Quality Improvement Project to Improve Lab Safety Monitoring in IBD Patients on Immunomodulators Using the Veterans Affairs IBD Dashboard and a Multidisciplinary Team. The American Journal of Gastroenterology. 2021;116(Sup 1):S458.

61. Hasan F., Pryce K., Puopolo G., Fiori K., Tomer G. Screening for social determinants of health in paediatric patients with inflammatory bowel disease in the outpatient setting: a quality improvement initiative. J Pediatr Gastroenterol Nutr. 2022;75(Supplement 1):S80.

62. Huggett, B, Chadokufa, S, Lindley, K, Shah, N, Kiparissi, F, Elawad, M, et al. Improvement of patient’s disease activity in paediatric inflammatory disease (pIBD) after adoption of Improve Care Now (ICN) quality improvement (QI) tool. Journal of Crohn’s and Colitis. 2014;8(Sup 2):S415–6.

63. Hyams K., Kennedy J., Green C., Mattoo S. A simple tool improves adherence to bone protection guidelines for inflammatory bowel disease. Gut. 2022;71(Supplement 1):A49–50.

64. Iqbal, Sara, Paine, Elizabeth. Immunization Rates in Inflammatory Bowel Disease Patients at the G.V. (Sonny) Montgomery VA Medical Center. American Journal of Gastroenterology. 2019;114(1):S1589–S1589.

65. Juakiem W, Gancayco J. Impact of Quality Improvement Interventions on Adherence to Quality Measures for Adults With Inflammatory Bowel Disease. Gastroenterology. 2018;155(1):e41–2.

66. Keith C, Kuo HC, Knight T, Maclin J, Jester T. Use of Quality Improvement Process to Increase Influenza Vaccination in Pediatric Inflammatory Bowel Disease Patients. Gastroenterology. 2018;154(1):S65.

67. Koumoutsos I, Kotha S, Warner B, Berry P. Setting up an integrated service for PSC-IBD patients: a quality improvement project. Gastroenterology Service. 2018;67(Sup 1):A245–6.

68. Lee, Ann, Eid, Emely. Providing fellows-in-training with education on inflammatory bowel disease health maintenance to improve the quality of care in our healthcare system. American Journal of Gastroenterology. 2014;109(Sup 2).

69. Lendner N., Perry S., Moses J., Sferra T., Young D. Standardizing steroid weaning protocols for patients with inflammatory bowel disease. J Pediatr Gastroenterol Nutr. 2022;75(Supplement 1):S419–20.

70. Levine AE, Chi LY, Sobczyk P, Bousvaros A, Trenor CC, Zitomersky N. Risk Stratification to Increase Venous Thromboembolism Prophylaxis in Pediatric Inflammatory Bowel Disease. Gastroenterology. 2019;156(3):S50–1.

71. Lin J., Yalan H., Chian L.W., Lin H., Liang R. Improving Influenza and Pneumococcal Vaccination Rates in an Inflammatory Bowel Disease Clinic at Tan Tock Seng Hospital, Singapore. Am J Gastroenterol. 2022;117(Supplement):S14.

72. Maisa, Abdalla, Jennifer, Lewis, Ashok, Shah, Lawrence, Saubermann, Thomas, Werth, Arthur, DeCross. The Performance of an Electronic Interactive Flow Sheet to Improve Preventive Health Care in Inflammatory Bowel Disease Patients. Inflammatory Bowel Diseases. 2014;20(Sup1):S57–9.

73. Mansoor S, Costantino J, Molle-Rios Z. Development of Care Pathway for Initiation of Enteral Nutrition Therapy for Pediatric Crohns Disease-Single Center Quality Improvement Initiative. Inflammatory Bowel Diseases. 2017;23(1):S74–5.

74. Mathew J, Thomas S, Samaha R, Prendaj E, Tomer G. The Influenza Vaccination and Pediatric Inflammatory Bowel Disease. Gastroenterology. 2019;156(6):S-616.

75. McConnell RA, Patel R, Sharpton SR, Velayos F, Mahadevan U. Improving the Quality of Inpatient Ulcerative Colitis Management: Promoting Evidence-Based Practice and Reducing Care Variation. Gastroenterology. 2017;152(5):S90–1.

76. Morency P., Bowditch S., Pawar A., Say D. Developing screening guidelines for thromboembolism prophylaxis in children with inflammatory bowel disease. J Pediatr Gastroenterol Nutr. 2022;75(Supplement 1):S74–5.

77. Navarro F., Rivera J., Turner D. Reliable data collection for quality improvement in children with inflammatory bowel disease. Small academic centre experience. J Pediatr Gastroenterol Nutr. 2022;75(Supplement 1):S80–1.

78. Pasquarella C, Frawley J, Raig K, Selvakumar PKC, Mahajan L. Naspghan nutrition prize quality improvement in pediatric inflammatory bowel disease: A successful strategy to improve disease-specific knowledge in patients and parents. Journal of Pediatric Gastroenterology and Nutrition. 2017;65(Sup 2):S210–1.

79. Patel N, Ali S, Burgis J, Stekol E, Isman C, Rodriguez L, et al. Approaching obesity in pediatric inflammatory bowel disease: a quality improvement initiative. Journal of pediatric gastroenterology and nutrition. 2021;73:s416–7.

80. Penninti, Pranav, Patel, Chirag, Coss, Elizabeth. Improving inflammatory bowel disease care: A quality improvement project for venous thromboembolism prophylaxis. American Journal of Gastroenterology. 2019;114.

81. Perlman M, Elmaoued R, Ganguli K, Israel E. Quality improvement: Using the PUCAI in the care of pediatric patients hospitalized with ulcerative colitis. Journal of Pediatric Gastroenterology and Nutrition. 2019;69(Sup 2).

82. Prendaj E, Thompson J, Tomer G. Improving Screening of Vitamin D Status in Pediatric Inflammatory Bowel Disease Patients. Gastroenterology. 2017;152(5):S435.

83. Raj, Priya, Ivanhoe, Lauren, Zbranek, Madilyn, Fegan-Bohm, Kelly. Implementing annual depression screening for adolescents with IBD using the PHQ-9A tool. Journal of Pediatric Gastroenterology and Nutrition. 69(Sup 2):2019.

84. Reich J, Zanchetti D, Wasan S, Miller H, Noronha A, Ardagna E, et al. A Quality Improvement Intervention Significantly Improved Influenza and Pneumococcal Vaccination Rates in Patients With Inflammatory Bowel Disease (IBD): 2206. Official journal of the American College of Gastroenterology. 2014;109:S640.

85. Rudra S, Grossman A, Downing M, Hillman J, Ashcroft K, Puma A, et al. Improving Influenza Vaccination in Pediatric Inflammatory Bowel Disease: A Quality Improvement Initiative. Gastroenterology. 2021;161(1):e32.

86. Saffouri G, Weber N, Singh S, Pardi D, Loftus Jr. EV, Kane S. Screening for bone health in patients with inflammatory bowel disease: A quality improvement project through point-of-care informational patient handouts. American Journal of Gastroenterology. 2015;110(Sup 1):S821.

87. Salem G, Ali IA, Grossen A, Bitar H, Kastens D. Bridges to Excellence (BTE) Quality Indicators in Inflammatory Bowel Disease (IBD) in Gastroenterology Fellows’ Clinic at the University of Oklahoma Health Sciences Center: A Quality Improvement Project. Gastroenterology. 2020;158(6):S-105-S-106.

88. Scherl EJ, Fajardo KI, Simone L, Carter J, Sapir T, Yang, Stevie, et al. A quality improvement initiative to reduce insurance-related delays in patient access to biologic therapies for inflammatory bowel disease. American Journal of Gastroenterology. 2019;114:S374–5.

89. Shakweh, Eathar, Middleton, Paul, Ahmad, Omer, Dart, Robin, McGuire, Joshua, Kader, Rawen, et al. An intervention bundle leads to quality improvement in endoscopic reporting of ulcerative colitis. Gut. 2021;70(Sup 1):A119–20.

90. Singh A., Midha V., Mahajan R., Verma S., Kakkar C., Grover J., et al. Evaluation of Nutritional Characteristics Reveals Similar Prevalence of Malnutrition in Patients with Ulcerative Colitis and Crohn’s Disease. Dig Dis Sci. 2023;68(2):580–95.

91. Smith CF, Lunn H, Wong G, Nicholson BD. Optimising GPs’ communication of advice to facilitate patients’ self-care and prompt follow-up when the diagnosis is uncertain: a realist review of ‘safety-netting’ in primary care. BMJ Qual Saf. 2021;31(7):541–54.

92. Subramanian S.K., Thomas A.R., Ramani A., Tsen A., Wadhwa V., Bhatt A., et al. Bone health monitoring and management in inflammatory bowel disease patients in the gastroenterology specialty clinic. Gastroenterology. 2022;162(7 Supplement):S-115.

93. Sussman L., Merdan O., Qualia C. Improving outcomes for pediatric patients with inflammatory bowel disease receiving biologic agents. J Pediatr Gastroenterol Nutr. 2022;75(Supplement 1):S235.

94. Ta A, Etter R, Berky S, Enderle L, Farrell P, Colman R, et al. Humira level 8 looking great: A quality improvement study on optimizing therapeutic drugmonitoring for patients with IBD on treatment with adalimumab (Humira). Journal of pediatric gastroenterology and nutrition. 2021;73:S414–5.

95. Talmadge C, Lewis G, Gold B, Gomez S, Reed B, Dykes D. Depression Screening in a Pediatric IBD Center. Gastroenterology. 2020;158(3):S101.

96. Tse CS, Elfanagely Y, Tanzer JR, Manudhane A, Rupawala A, Fine SD. Reduction of Chronic Steroid Use in Patients Discharged for Inflammatory Bowel Disease Flares: A 28-Month Quality Improvement Study. Gastroenterology. 2021;161(1):e33.

97. Turner, D, Nehemia, C, Yerushalmy-Feler, A, Assa, A, Slae, M, Kori, M, et al. A nationwide quality improvement program in children with Crohn’s disease improves outcomes within 12 months. Journal of Crohn’s and Colitis. 2019;13(Sup 1):S361.

98. Viganò C, Meucci G, Saibeni S, Cortelezzi CC, Amato A. Quality in IBD Care: Measure, Educate and Improve. A Real-Life Survey. Gastroenterology. 2016;150(4):S800.

99. Walker T, Thomas A, Cushing K, Bennett M, Badillo R, Sayuk G, et al. Improving Vaccination Adherence in Inflammatory Bowel Diseases. Gastroenterology. 2019;157(1):e35.

100. Walker T, Thomas A, Wade FG, Jacquez J, Ciorba MA, Deepak P, et al. Enhancing Utilization of Therapeutic Drug Monitoring in Inflammatory Bowel Disease Correlates with Improved Outcomes. Gastroenterology. 2019;156(6):S-1140-S-1141.
