# Supplementary material for: Quality improvement exercises in Inflammatory Bowel Disease (IBD) services: A scoping review
Source: PLoS One. 2024 Mar 7;19(3):e0298374. doi: 10.1371/journal.pone.0298374 (PMC10919633; doi:10.1371/journal.pone.0298374)
Supplement: Attachment — Submitted filename: Response to Reviewers.docx [file pone.0298374.s005.docx]

Dear Dr Pittet,

We would like to express our thanks to you and the reviewer for reviewing our manuscript. We appreciate the consideration of our paper, and the detailed response provided. The comments given were insightful, and by addressing these we believe that we have improved the quality of this report. Please see details on how we have addressed the reviewer comments below:

- Use of the IBD standards: The IBD standards are not NHS standards, but were developed by IBD UK. We believe these are useful for categorisation as they describe the stages of the patient journey, allowing us to highlight areas of the journey with fewer published quality improvement papers. However, we agree that the definition of these required more explanation, therefore have defined these in the introduction (page 3) and further expanded on this in the methods (pages 7 to 8).
- Grouping of anaemia, PSC and obesity in stratified care: PSC and obesity have been grouped here as these two papers focus on specific subsets of IBD patients – screening/treatment specific to those with additional conditions. However, after consideration, the anaemia article has been re-categorised into ‘Screening’ as this did focus on the whole population.
- Description of unsuccessful studies: As mentioned by the reviewer, due to publication biases, almost all articles report some level of success, leaving little insight into unsuccessful interventions. As we have now added results to Table 2 and S4, it should be clearer how successful the individual interventions were.
- Expansion of Table 2: We have added more details on the intervention components and the outcomes/results for all papers. Though we attempted to structure as suggested, with intervention categories in separate rows alongside outcomes, this looked very unclear, as individual articles used multiple interventions. However, by adding the details, and by adding an extra ‘overarching theme’ column, we believe this more clearly shows what is being improved and how it was accomplished, allowing the table to serve as a standalone if necessary.
- Expansion of S4: Similarly to table 2, we have added intervention components and outcomes to S4 to provide more summaries of the research.
- Translation of results into take-home messages: We have added overarching themes to Table 2, and added further thoughts to the discussion.
- Limitation of search strategy: We have added this the limitations section.

Finally, as requested, I have updated our statement regarding competing interests, as follows. Thank you for changing this in our online submission form: *I have read the journal's policy and the authors of this manuscript have the following competing interests: Professor Alan Lobo has acted as a consultant and advisory board member for Takeda Pharma, Janssen and Bristol Myers Squibb. This does not alter our adherence to  PLOS ONE policies on sharing data and materials.*

Again, thank you for your consideration of our manuscript, and we look forward to hearing from you at your earliest convenience.

Sincerely,

Katie Ridsdale
